# Preliminary Assessment of Post-traumatic Stress Disorder Symptoms Among Emergency Medicine Physicians During the COVID-19 Outbreak

**DOI:** 10.1016/j.acepjo.2025.100098

**Published:** 2025-04-03

**Authors:** Sriram Venkatesan, Arthi Kozhumam, Eleanor Strand, Catherine A. Staton, Sreeja M. Natesan, João Ricardo Nickenig Vissoci, John David Purakal

**Affiliations:** 1 Sri Ramachandra Medical College & Research Institute, Chennai, India; 2 Medical Scientist Training Program, Northwestern University Feinberg School of Medicine, Chicago, Illinois, USA; 3 Rural Health Research Center, University of Minnesota – Twin Cities, Minneapolis, Minnesota, USA; 4 Global Emergency Medicine Innovation and Implementation Research Center (GEMINI), Duke University, Durham, North Carolina, USA; 5 Department of Emergency Medicine, Duke University School of Medicine, Durham, North Carolina, USA; 6 Duke Global Health Institute, Durham, North Carolina, USA; 7 Division of Translational Health Sciences, Department of Emergency Medicine, Duke University School of Medicine, Durham, North Carolina, USA; 8 Research Design and Analysis Core (RDAC), Duke Global Health Institute, Duke University, Durham, North Carolina, USA; 9 Duke-Margolis Institute for Health Policy, Duke University, Durham, North Carolina, USA

**Keywords:** infectious diseases, COVID-19, physician wellness, post-traumatic stress disorder, emergency physicians

## Abstract

**Objectives:**

The COVID-19 pandemic has caused significant increases in work-related emotional stress and emergency department (ED) volumes. Our study aimed to provide a preliminary assessment of posttraumatic stress disorder (PTSD) symptoms among emergency medicine (EM) physicians in the United States following the COVID-19 pandemic and explore related factors and predictors of PTSD symptoms.

**Methods:**

Participants were recruited using a convenience sampling approach via professional listservs from national and state EM societies. Eligible participants included board-certified or board-eligible EM physicians, EM residents, and non-EM physicians working in an EM setting during the pandemic. The survey was distributed online using Qualtrics, ensuring anonymity and data security, from September 2020 to April 2021, with active recruitment during 2 periods: September to October 2020 and March to April 2021. To optimize clarity, the survey was prepiloted and measures were included to prevent duplicate responses. Descriptive analyses were reported with percentages, means, and medians using RStudio, PBC.

**Results:**

A total of 362 surveys were distributed, of which 315 (87%) were completed and included in the analysis. Participants were predominantly aged 35 to 50 years (46%), White (86%), and board-certified in EM (70%), with most practicing in urban level 1 trauma centers (45%). Geographic representation included 40 states, with the largest proportions from the South (33%) and Midwest (29%). Overall, 92% of participants reported experiencing PTSD symptoms, with 41% classified as minimal, 22% mild, 18% moderate, 12% severe, and 7% very severe. The median PTSD Symptom Scale (PSS-I-5) score was 13 (IQR, 23). Factors associated with more severe PTSD symptoms included prior mental health diagnoses, female sex, and working in rural settings.

**Conclusions:**

The study highlights the widespread occurrence of PTSD symptoms, with 92% of EM physicians reporting symptoms during the COVID-19 pandemic. Demographic and workplace factors, such as prior mental health diagnoses, female sex, and rural practice settings, contributed to greater symptom severity. These findings underscore the need for targeted mental health interventions and resources tailored to the specific needs of this at-risk group.

**The Bottom Line**

Emergency medicine (EM) physicians faced immense psychological stress during the COVID-19 pandemic, with prevalence and severity of posttraumatic stress disorder symptoms in this group remaining underexplored. This study found that 92% of EM physicians experienced posttraumatic stress disorder symptoms, with 41% classified as minimal, 22% mild, 18% moderate, 12% severe, and 7% very severe. The severity of symptoms was influenced by factors such as prior mental health diagnoses, female sex, and practicing in rural settings. These findings highlight the critical need for mental health interventions tailored to emergency physicians, especially during large-scale crises, to safeguard the well-being of this essential workforce.

## 1 Introduction

### 1.1 Background

Emergency medicine (EM) physicians are regularly confronted with hectic work conditions and work-related traumatic events. Emergency departments (EDs) are highly dynamic and stressful care environments, where physicians are responsible for a variety of patients with illnesses of varying acuities. Due to these factors, EM physicians are already under considerable stress during their normal work hours. In the pre-COVID-19 era, posttraumatic stress disorder (PTSD) was demonstrated to be prevalent (11.9%-16.8%) among EM physicians compared with other specialties, with triggers including pediatric or unexpected deaths, mass casualty events, staff injury/death, handling burn victims, and confrontations with psychiatric patients.^1^, ^2^, ^3^, ^4^, ^5^, ^6^, ^7^

The magnitude of the impact of mass casualty outbreaks on the mental health of frontline health care professionals has been well recognized.^8^ Initial COVID-19 studies in China with frontline health care workers reported high levels of anxiety and PTSD-related symptoms including more than 27% with stress symptoms and 23% with anxiety symptoms, or 40% screening positive for PTSD symptoms.^9^^,^^10^ A similar study conducted on EM physicians in the United States, during the initial few months of the pandemic, between April and June 2020, revealed that 22% showed symptoms of stress consistent with PTSD.

### 1.2 Importance

With the growing number of health care professionals suffering the mental health impact of COVID-19 worldwide working in overburdened, overworked, and underequipped health care systems, it is imperative to learn more about the incidence of PTSD among EM physicians during the pandemic in the United States, factors contributing to PTSD in this group, and effective strategies to combat them to prepare for future pandemics.^11^

### 1.3 Goals of This Investigation

Our study aimed to provide a preliminary assessment of PTSD symptoms among EM physicians in the United States following the COVID-19 pandemic.

## 2 Methods

### 2.1 Study Design

This was a repeated cross-sectional (RCS) study in which US-based EM physicians were invited to complete an online, self-report survey at 2 time points: September to October 2020 and March to April 2021, corresponding to the first and second waves of the COVID-19 pandemic. The RCS design minimized participant burden while allowing for valid inference of changes over time.

Ethical approval was obtained from the Duke University Health System Institutional Review Board (Pro00106210). The study adhered to the consensus-based checklist for reporting of survey studies (CROSS) to enhance transparency and quality in reporting.^12^

### 2.2 Selection of Participants

The study was conducted across the United States, with responses collected from participants representing 40 states, including the South (33%), Midwest (29%), Northeast (21%), and West (17%).

Eligible participants included the following:

1. Board-certified or Board-eligible EM physicians,
2. EM residents, and
3. Non-EM physicians working in Level 1/2/3/4 trauma centers during the COVID-19 pandemic.

Participants were excluded if they were over the age of 65 years or working in an urgent care center to ensure sufficient sample size for analysis. A 2-tier convenience sampling method was employed to optimize reach. Verified national and state EM organizations were contacted to distribute the survey via listservs or affiliated social media groups. Recruitment occurred at the start of each survey period, with 3 biweekly reminders sent to organizations. Participation was voluntary, and implied consent was obtained upon survey completion.

The survey was sent to members across several professional listservs, including the American College of Osteopathic Emergency Physicians (ACOEP, approximately 3000 members), the American College of Emergency Physicians (ACEP Members Listserv, 36,700 members), the Emergency Medicine Residents' Association (EMRA Listserv, 16,000 members), and the Society for Academic Emergency Medicine (SAEM Listserv, 8000 members). All state ACEP chapters, including the Florida College of Emergency Physicians, Arizona ACEP, and West Virginia College of Emergency Physicians, denied disclosing their membership numbers. The survey was also shared on EMDocs.net, a free, open-access medical education website for EM physicians, although no analytics are available to assess its reach. Despite efforts to calculate an accurate response rate, the lack of data on recipients who accessed the survey from these listservs and EMDocs.net made this calculation infeasible. However, the total known membership from disclosed listservs exceeds 63,700 individuals.

The survey was administered online using Qualtrics, a secure platform for web-based research. All response data were stored securely on Qualtrics, with access limited to study personnel. To prevent duplicate responses, the Qualtrics Relevant ID function was used to analyze metadata and flag potential duplicates; no duplicate responses were identified.^13^

The sample size was calculated based on an estimated PTSD prevalence of 27% (from prior studies of health care workers during the pandemic), with a 95% CI (± 5%).^9^^,^^10^ The target was 302 completed surveys.^14^

### 2.3 Measurements

The survey consisted of 43 items, which is divided into the following sections:

1. Mental health history (5 items): Previous mental health diagnoses and current mental health services.
2. PTSD symptoms (20 items): Measured using the PTSD Symptom Scale (PSS-I-5), supplemented with a perceived stressor item.^15^
3. Demographics (9 items): Age, gender, race, geographic location, and practice setting.
4. Short Form-8 (SF-8) instrument (8 items): General health and well-being assessment.^16^

The PSS-I-5 is a validated, semi-structured tool for assessing PTSD symptoms based on Diagnostic and Statistical Manual of Mental Disorders, 5th ed. (DSM-5) criteria, measuring 4 domains: re-experiencing, avoidance, increased arousal/reactivity, and changes in cognition/mood. Scores range from 0 to 80, categorized as follows:

1. Minimal (0-8)
2. Mild (9-18)
3. Moderate (19-30)
4. Severe (31-45)
5. Very severe (>45)

We have innovatively used the American Psychological Association–approved PSS-I-5 tool, to assess the severity of the PTSD symptoms in EM physicians. For this study, the tool was adapted for self-reporting to improve accessibility. Modifications included the following:

1. Using the “past month” version to evaluate PTSD symptom severity over the pandemic.
2. Asking participants to base responses on their cumulative experiences since February 2020 rather than a single traumatic event.
3. Administering the survey online instead of via in-person interviews.

These changes were made in consultation with the developers of the PSS-I-5 to minimize validity concerns.^15^

The survey was pretested among 5 EM physicians to ensure clarity and usability.

### 2.4 Data Analyses

Only fully completed surveys were included in the analysis. Two surveys were excluded due to incomplete responses in the SF-8 section.

Descriptive statistics were used to summarize mental health history, demographics, and PTSD symptom severity categories. Differences in PSS-I-5 scores and demographic variables were assessed using Pearson’s chi-squared test, Fisher’s exact test, and Wilcoxon rank-sum test.

Statistical significance was set at *P* < .05. Data analysis and visualization were performed using RStudio, PBC (v. 4.1.1).^17^

Response rates could not be calculated due to the 2-tier sampling strategy, as organizations distributed the survey link without tracking the number of recipients.

## 3 Results

### 3.1 Participant Characteristics

Of 362, 315 (87%) surveys initiated by participants were submitted. A total of 103 (33%) surveys were submitted in the first cross-section between September and October 2020, and 212 (67%) were submitted in the second between March and April 2021 (Table 1). There were more male participants (73%) and participants with a previous mental health diagnosis (75%) in the second cross-section. There were no statistically significant differences between the cross sections in the other demographic variables.

**Table 1:** Participant demographics by cross-section.

Participant characteristics	All participants	Sept-Oct 2020	March-April 2021	*P*
	N = 315^a^	N = 103^a^	N = 212^a^	
Age (y)				.8
20-35	94 (30%)	31 (33%)	63 (67%)	
35-50	144 (46%)	46 (32%)	96 (68%)	
50-65	67 (21%)	25 (37%)	42 (63%)	
Sex				.003
Female	134 (43%)	56 (43%)	74 (57%)	
Male	181 (57%)	46 (27%)	127 (73%)	
Race				>.9
Caucasian/White	272 (86%)	88 (34%)	173 (66%)	
Asian	14 (4.4%)	5 (36%)	9 (64%)	
African American/Black	9 (2.9%)	3 (38%)	5 (62%)	
Hispanic/Latino	7 (2.2%)	2 (29%)	5 (71%)	
Preferred not to answer	13 (4.1%)	4 (31%)	9 (69%)	
Military service				.3
Veteran or active military	27 (8.6%)	6 (25%)	18 (75%)	
Position				.4
EM board-certified	219 (70%)	74 (36%)	134 (64%)	
EM Resident	60 (19%)	15 (25%)	45 (75%)	
EM Board-eligible	31 (9.8%)	12 (39%)	19 (61%)	
Non-EM board-certified	5 (1.6%)	1 (25%)	3 (75%)	
Mental health history				
Previous PTSD diagnosis	14 (4.4%)	3 (23%)	11 (77%)	.6
Previous mental health diagnosis	84 (27%)	20 (25%)	64 (75%)	.046
Currently receiving mental health services	69 (22%)	19 (28%)	50 (72%)	.2

Workplace characteristics	N = 315^a^	N = 103^a^	N = 212^a^	
EM setting				.085
Level 1 trauma center	141 (45%)	57 (41%)	82 (59%)	
Level 2 trauma center	58 (18%)	13 (24%)	41 (76%)	
Level 3/4 trauma center	78 (25%)	22 (29%)	54 (71%)	
Other	36 (11%)	10 (29%)	24 (71%)	
Population served				.15
Urban	171 (54%)	64 (38%)	103 (62%)	
Suburban	102 (32%)	28 (29%)	68 (71%)	
Rural	42 (13%)	10 (25%)	30 (75%)	
Region				–
South	104 (33%)			
Midwest	91 (29%)			
West	56 (17%)			
Northeast	64 (21%)			

EM, emergency medicine; PTSD, posttraumatic stress disorder.

Significance is defined as a *P* value less than .05.

a n (%).

Most participants were between age 20 and 50 years (76%), Caucasian/White (86%), EM board-certified (70%), and worked in a level 1 or 2 trauma center (63%). There were slightly more male participants than female (57%). About 1 in 4 participants had a previous mental health diagnosis, and 1 in 5 were currently receiving mental health services. A total of 14 participants (4.4%) had a previous diagnosis of PTSD. Of these 14, three were active military or veterans and half were currently receiving mental health services.

Participants were roughly distributed across the United States, representing 40 of 50 states across the South (33%), Midwest (29%), Northeast (21%), and West (17%). States with the most participants were Florida (9.5%), New York (8.6%), California (7.6%), Ohio (7.6%), Arizona (7.0%), Texas (6.7%), and Michigan (6.0%).

### 3.2 PSS-I-5 Scores

Despite efforts to calculate an accurate response rate, the lack of data on recipients who accessed the survey from these listservs and EMDocs.net made this calculation infeasible. However, the total known membership from disclosed listservs exceeds 63,700 individuals.

The median was 13 (IQR, 23) in all participants. According to the severity scale in the PSS-I-5 manual, 41% of participants experienced PTSD symptoms at minimal severity, 22% at mild, 18% at moderate, 12% at severe, and 7% at very severe. There was no statistically significant difference in the traumatic experience and PSS-I-5 variables across cross sections (Table 2).

**Table 2:** Trauma experience and PSS-I-5 across cross-section.

Characteristic	Total Participants (N = 313)^a^	Sept-Oct 2020 (N = 103)^a^	March-April 2021 (N = 210)^a^	*P*^b^
DSM-5 traumatic experience				.11
Reporting experiencing trauma at work because of COVID-19	174 (56%)	51 (50%)	125 (59%)	
PSS-I-5 raw score				.70
Median (IQR)	13 (23)	11 (22)	13 (23)	
PSS-I-5 severity category				>.9
Minimal (0-8)	127 (41%)	44 (43%)	83 (39%)	
Mild (9-18)	68 (22%)	21 (20%)	49 (23%)	
Moderate (19-30)	57 (18%)	18 (17%)	39 (18%)	
Severe (31-45)	39 (12%)	13 (13%)	26 (12%)	
Very severe (46-80)	22 (7.0%)	7 (6.8%)	15 (7.1%)	

DSM, Diagnostic and Statistical Manual; PSS, Posttraumatic Stress Disorder Symptom Scale.

a n (%).

b Pearson's chi-squared test; Wilcoxson rank-sum test (between cross sections).

All participants reported feeling at least once source of stress in their workplace, with 244 (77%) reporting multiple. Major sources of stress were fear of themselves (47%) or their family/friends getting sick (61%), shift acuity (50%), overcrowding (48%), lack of a standard for treating COVID-19 (32%), and lack of personal protective equipment (PPE) (26%) (Table 3). Participants noted a “systematic lack of caring” from hospital administration, watching “sad patients dying alone because of visitor restrictions,” and working in a “makeshift morgue.”

**Table 3:** Selected sources of traumatic stress (participants were able to select multiple).

Source of stress	Total participants (N = 315)^a^
Fear of family/friends getting sick	192 (61%)
Shift acuity	158 (50%)
Overcrowding	151 (48%)
Fear of getting sick	147 (47%)
Lack of standard for treating COVID-19	107 (32%)
Lack of personal protective equipment	81 (26%)
Other (free text entry)	
Experiencing death and dying	10 (3%)
Feeling of blame/lack of support from hospital administration	9 (2.8%)
COVID misinformation/politicalization	7 (2%)
Fear of losing their job/income	2 (0.6%)
COVID-19 is not a source of stress	2 (0.6%)

a n (%).

The variables sex, previous mental health diagnosis, mental health services, and population served were associated with PTSD symptom severity (Table 4). A total of 72% of female participants had mild, moderate, severe, or very severe symptoms vs 50% of males. Participants with a previous mental health diagnosis and/or who were currently receiving mental health services had more severe PTSD symptoms than those without a diagnosis and/or who were not receiving services. Lastly, participants who served a rural population had more severe PTSD symptoms than those who served urban or suburban populations.

**Table 4:** Association of participant and workplace characteristics with PTSD symptom severity.

Participant characteristics	Minimal (0-8), N = 122^a^	Mild and moderate (9-30), N = 122^a^	Severe and very severe (>30), N = 59^a^	*P*^b^
Age (y)				.4
20-35	43 (46%)	33 (35%)	18 (19%)	
35-50	54 (38%)	57 (40%)	31 (22%)	
50-65	25 (37%)	32 (48%)	10 (15%)	
Sex				<.001
Female	36 (28%)	61 (47%)	33 (25%)	
Male	86 (50%)	61 (35%)	26 (15%)	
Race				.7
Caucasian/White	103 (39%)	107 (41%)	51 (20%)	
Asian	6 (43%)	6 (43%)	2 (14%)	
African American/Black	6 (75%)	1 (12%)	1 (12%)	
Hispanic/Latino	3 (43%)	3 (43%)	1 (14%)	
Preferred not to answer	4 (31%)	5 (38%)	4 (31%)	
Military service				.5
Veteran or active military	11 (46%)	7 (29%)	6 (25%)	
Position				.8
EM board-certified	83 (40%)	84 (40%)	41 (20%)	
EM board-eligible	14 (45%)	11 (35%)	6 (19%)	
EM resident	24 (40%)	26 (43%)	10 (17%)	
Non-EM board-certified	1 (25%)	1 (25%)	2 (50%)	
Mental health history				
Previous PTSD diagnosis	2 (15%)	9 (69%)	2 (15%)	.088
Previous mental health diagnosis	19 (23%)	38 (47%)	24 (30%)	<.001
Currently receiving mental health services	16 (23%)	29 (42%)	24 (35%)	<.001


Workplace characteristics	N = 122^a^	N = 122^a^	N = 59^a^	
EM setting				.3
Level 1 trauma center	60 (43%)	58 (42%)	21 (15%)	
Level 2 trauma center	21 (39%)	19 (35%)	14 (26%)	
Level 3/4 trauma center	25 (33%)	31 (41%)	20 (26%)	
Other	16 (47%)	14 (41%)	4 (12%)	
Population served				.027
Rural	13 (32%)	12 (30%)	15 (38%)	
Suburban	36 (38%)	41 (43%)	19 (20%)	
Urban	73 (44%)	69 (41%)	25 (15%)	

EM, emergency medicine; PTSD, posttraumatic stress disorder.

Significance is defined as a *P* value less than .05.

a n (%).

b Pearson's chi-squared test; Fisher's exact test.

## 4 Limitations

This study has multiple limitations. First, recruitment via professional association listservs may have introduced sampling bias, as physicians less engaged with these associations might not have participated. Although the survey was distributed to several professional listservs, state chapters of the ACEP did not disclose their membership numbers. This limited our ability to estimate the total reach of the survey accurately. Additionally, the listservs did not track how many members received or accessed the survey link, making it impossible to calculate an accurate response rate. These factors may affect the generalizability of our findings.

The reliance on self-reported data carries risks of recall and social desirability biases, potentially affecting the accuracy of the results. Moreover, the study's cross-sectional design, with data collected at only 2 time points, limits its ability to analyze longitudinal trends in PTSD symptom development. Expanding future studies to include multiple time points could provide valuable insights into symptom progression over time. Furthermore, the voluntary nature of survey participation may have introduced selection bias, potentially skewing the results toward those experiencing more severe PTSD symptoms. Strategies to incentivize broader participation could mitigate this issue in future research.

Finally, although participants were geographically diverse, certain subgroups, such as rural physicians and minority groups, were underrepresented, which may limit the generalizability of our findings. Future studies should incorporate stratified sampling or oversampling of underrepresented groups to ensure comprehensive coverage.

## 5 Discussion

Work stress is a threat to an EM physician’s wellness, especially during prolonged periods of traumas experienced by health care systems during the pandemic. We examined the widespread occurrence of PTSD symptoms in frontline EM physicians across the United States, during the first, second, and third waves of the COVID-19 pandemic between September 2020 and April 2021. Our innovative cross-sectional survey of EM physicians at 2 time periods—during the first wave of the pandemic vs second wave found 92% of EM physicians suffered PTSD symptoms, with no changes in PSS-I-5 scores reported later in the pandemic (Figure). Primary stressors included shift acuity, overcrowding, fear of getting sick, fear of family and friends getting sick, lack of PPE, and dissatisfaction with hospital administration.

**Figure:**
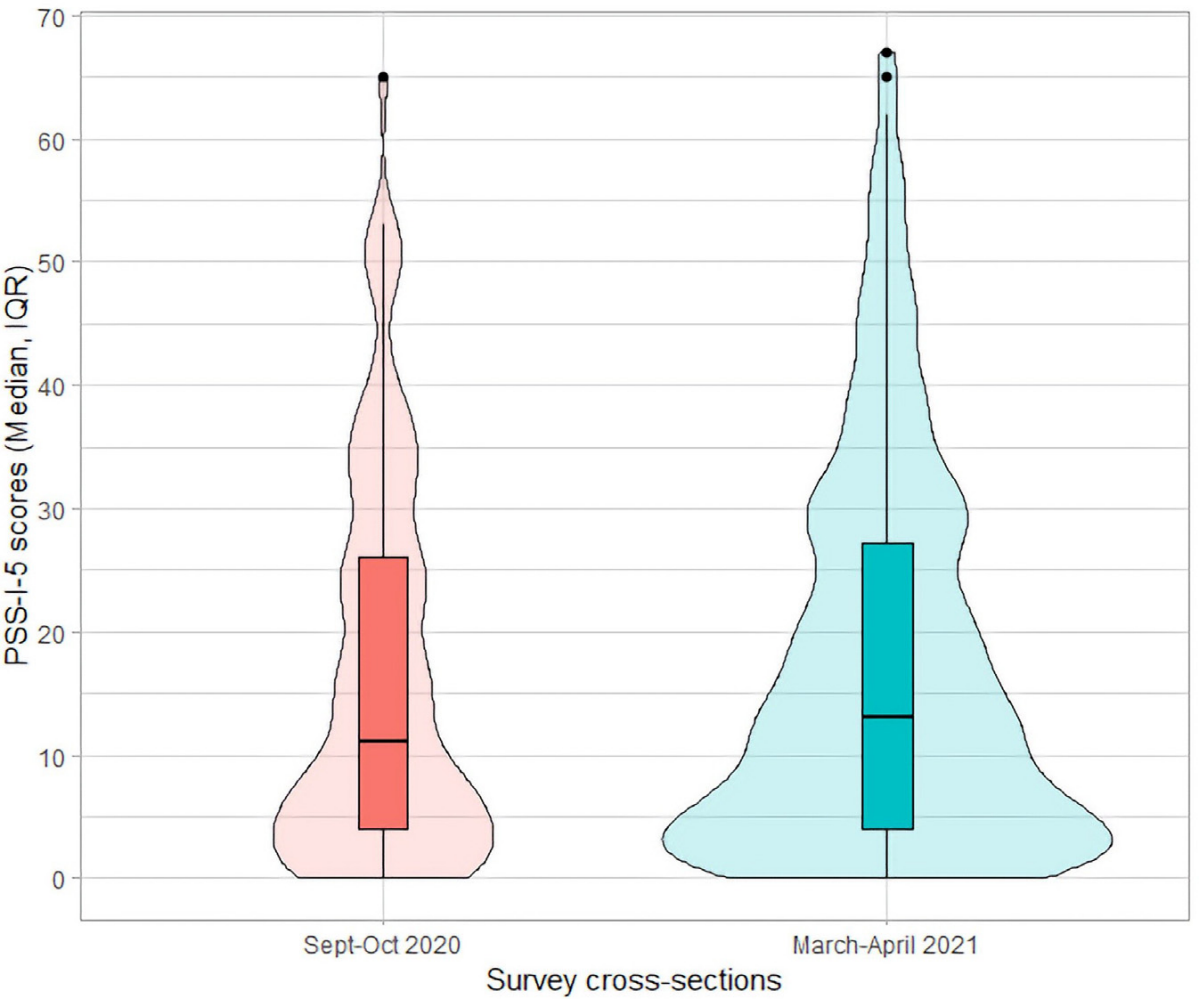
PSS-I-5 score distribution by cross-section. PSS, Posttraumatic Stress Disorder Symptom Scale.

The concepts of burnout and coping strategies for stress in EM have been studied for decades. Medical professionals exposed to extraordinary conditions of suffering have often shown to be at a heightened risk for conditions like PTSD.^1^ The types of physicians most prone to developing PTSD are physicians who practice EM in rural areas with limited resources, those in residency training, those who are involved in malpractice litigation, and/or those who are indirectly exposed to trauma.^7^ As reported in our study, the baseline stressors of EM coupled with the added burdens brought on by acute pandemics like the COVID-19 pandemic can have a great impact on an EM physician’s mental state of mind. Our study also emphasized how PTSD symptom severity was greatly affected by a history of past mental health diagnoses and the population the physician served. This finding correlates well with other past studies, including a cross-country study across Egypt, Germany, and Italy which found participants with a history of past exposure to traumatic events as being at an increased risk of developing PTSD with psychopathological symptoms.^18^ Regarding the gender differences, our study showed that these symptoms were higher among the female participants. This is consistent with previous studies, which show women are 2 times more likely than men to develop PTSD symptoms. Another study on Australian frontline health care workers that showed rural workers experiencing a higher prevalence of mental health symptoms despite treating very few COVID-19 patients during this period, correlated with our study results.^19^

According to the 2020 National Study of the Emergency Physician Workforce, there were 48,835 clinically active EM physicians in 2020. Most EM physicians practiced in urban areas (92%), whereas 2730 (6%) practiced in large rural areas and 1197 (2%) in small rural areas.^20^ Our study heavily aligns with this data because majority of our participants also practiced in urban settings.

Our results highlight the profound impact that COVID-19 has had on the mental health of professionals on the frontline, specifically in the ED. Our study included a large number of participant members from several EM organizations including the ACEP, EMRA, SAEM, and Council of Residency Directors in Emergency Medicine, which we feel is a significant strength toward the application of this knowledge. The conclusions of our study, in relation to baseline stress factors and the added burdens brought by acute mass casualty events like the COVID-19 pandemic, also suggest the generalizability of our observations. Our study is also notably geographically diverse, with respondents from 40 states. It is also the first study to employ this scale to (1) broadly identify the widespread occurrence of PTSD symptoms in EM physicians in the United States and (2) analyze the severity along with factors contributing to these symptoms in EM physicians.

Our findings further demonstrate the need to prioritize mental health for EM physicians and make resources available to them, especially during periods of widespread increased stress, such as institutional hardships or pandemic-type events. The Centers for Disease Control and Prevention has made recommendations for employers in response to the COVID-19 pandemic, which call for an effective response plan incorporating factors like healthy work conditions, improved leave policies, and resilience-building among workers.^21^ Today, 4 years after the start of the COVID-19 pandemic, stressors continue to mount for EM physicians, with rising rates of workplace violence, staffing shortages, and ED boarding.^22^, ^23^, ^24^ Despite an increase in mental health awareness for physicians over that period, wellness initiatives and the aforementioned mental health support services for frontline responders are still inconsistently available, with limited access to employee assistance programs, and few emergency physicians seeking external treatment outside the organization.^22^ Our study adds to the body of literature highlighting how the COVID-19 pandemic exacerbated mental health issues among EM physicians. It further spotlights the urgent need for sustained and rejuvenated focus on the well-being of EM physicians, along with providing adequate mental health resources and support for our high-risk cohort.

## Author Contributions

SV and JDP conceived the study. SV, JDP, CAS, and SMN designed the study plan. SV and JDP supervised data collection. SV managed collected data, and quality control. AK, ES, JRNV, and CAS provided statistical advice on study design and analyzed the data. SV drafted the manuscript, and all authors contributed substantially to its revision.

## Funding and Support

By *JACEP Open* policy, all authors are required to disclose any and all commercial, financial, and other relationships in any way related to the subject of this article as per ICMJE conflict of interest guidelines (see www.icmje.org). The authors have stated that no such relationships exist.

## Conflict of Interest

All authors have affirmed they have no conflicts of interest to declare.
