# Supplementary material for: Preliminary Assessment of Post-traumatic Stress Disorder Symptoms Among Emergency Medicine Physicians During the COVID-19 Outbreak
Source: J Am Coll Emerg Physicians Open. 2025 Apr 3;6(3):100098. doi: 10.1016/j.acepjo.2025.100098 (PMC11999589; doi:10.1016/j.acepjo.2025.100098)
Supplement: Supplementary Material [file mmc1.pdf]

TRAUMA SCREEN

Many people are exposed to a disturbing or traumatic event at some point in their lives. These experiences can happen in any of the following ways:

1. Directly experiencing the event
2. Witnessing the event
3. Learning that the event happened to a close family member or close friend
4. Experiencing repeated or intense exposure to distressing details of the event (e.g. emergency workers collecting human remains)

Examples of traumatic events include: natural disasters, accidents, sexual assaults, physical assaults, combat, childhood sexual abuse, torture, or life-threatening illness.

Have you experienced such an event?

___Yes
___No

Please briefly describe the experience which is the **most distressing and the most haunting for you currently**.

If you are unsure, briefly describe the experience anyway:

____

____

____

____

Did this event included:

a. Actual or threatened death? Yes No
b. Actual or threatened serious injury? Yes No
c. Actual or threatened sexual violation Yes No

When did this event occur? ____

©2013 Edna B. Foa
All rights reserved. Reproduction or redistribution, in whole or in part, in any manner, without the prior written consent of the copyright holder, is a violation of copyright law.

Mental health researchers and clinicians may make copies of the measure for their own clinical and research use. Any other use is prohibited.

PSS-I-5

Questions should be about the most currently distressing trauma. Each item below should be asked in reference to the past month (if < 1 month since trauma, ask "Since the event…"). Probe all positive responses (e.g., "How often has this been happening?") following the instructions provided in the PSS-I-5 manual.

0	1	2	3	4
Not at all	Once a week or less/a little	2 to 3 times a week/somewhat	4 to 5 times a week/very much	6 or more times a week/severe

RE-EXPERIENCING (need one): [probe, then quantify]

____ 1. Have you had unwanted distressing memories about the trauma?

____ 2. Have you been having bad dreams or nightmares related to the trauma?

____ 3. Have you had the experience of feeling as if the trauma were actually happening again?

____ 4. Have you been very EMOTIONALLY upset when reminded of the trauma?

____ 5. Have you had PHYSICAL reactions when reminded of the trauma (e.g., sweating, heart racing)?

AVOIDANCE (Need one): [probe, then qualify]

____ 6. Have you been making efforts to avoid thoughts or feelings related to the trauma?

____ 7. Have you been making efforts to avoid activities, situations, or places that remind you of the trauma or that feel more dangerous since the trauma?

CHANGES IN COGNITION AND MOOD (Need two): [probe, then qualify]

____ 8. Are there any important parts of the trauma that you cannot remember?

____ 9. Have you been viewing yourself, others, or the world in a more negative way (e.g."I can't trust people," "I'm a weak person")?

____10. Have you blamed yourself for the trauma or for what happened afterwards? Have you blamed others that did not directly cause the event for the trauma or what happened afterwards?

____11. Have you had intense negative feelings such as fear, horror, anger, guilt or shame?

____12. Have you lost interest in activities you used to do?

____13. Have you felt detached or cut off from others?

____14. Have you had difficulty experiencing positive feelings?

0	1	2	3	4
Not at all	Once a week or less/a little	2 to 3 times a week/somewhat	4 to 5 times a week/very much	6 or more times a week/severe

INCREASED AROUSAL AND REACTIVITY (need two): [probe then quantify]

____15. Have you been acting more irritable or aggressive?

____16. Have you been taking more risks or doing things that might cause you or others harm (e.g., driving recklessly, taking drugs, having unprotected sex)?

____17. Have you been overly alert or on-guard (e.g., checking to see who is around you, etc.)?

____18. Have you been jumpier or more easily startled?

____19. Have you had difficulty concentrating?

____20. Have you had difficulty falling or staying asleep?

TOTAL SCORE (add items 1-20) = _______

DISTRESS AND INTERFERENCE

____21. How much have these difficulties been bothering you?

____22. How much have these difficulties been interfering with your everyday life (e.g. relationships, work, or other important activities)?

SYMPTOM ONSET AND DURATION

23. How long after the trauma did these difficulties begin? [circle one]
    a. Less than 6 months
    b. More than 6 months

24. How long have you had these trauma-related difficulties? [circle one]
    a. Less than 1 month
    b. More than 1 month
