# Supplementary material for: Preliminary Assessment of Post-traumatic Stress Disorder Symptoms Among Emergency Medicine Physicians During the COVID-19 Outbreak
Source: J Am Coll Emerg Physicians Open. 2025 Apr 3;6(3):100098. doi: 10.1016/j.acepjo.2025.100098 (PMC11999589; doi:10.1016/j.acepjo.2025.100098)
Supplement: Supplementary Material [file mmc2.docx]

**Copy of Survey Tool Used**

Research Survey Study: PTSD Symptoms in ED Physicians during COVID-19

Hello,

We are looking for physicians working in Emergency Departments across the United States to participate in a research study entitled “A Survey of PTSD Symptoms and its Predictors among Emergency Physicians in the United States during the COVID-19 Outbreak.” Participation in this study is voluntary.

Eligibility Criteria:

- US Board-Certified/Board-Eligible Emergency Medicine Physician
- Emergency Medicine Resident in the United States
- US Non-EM Physician working in an EM setting

The purpose of this study is provide information about the prevalence of Post-Traumatic Stress Disorder (PTSD) symptoms among Emergency Medicine physicians nationwide in the United States following the COVID-19 pandemic, and to explore other related factors including predictors of these symptoms.

If you agree to participate, you will be asked to complete a survey about your experiences as an Emergency Room physician during the COVID-19 pandemic. This survey may contain questions that are sensitive or upsetting. You may skip any questions that make you uncomfortable or that you do not wish to answer. No information that can identify you will be collected and your name and contact information will not be linked with the survey results. This survey should take you about 10 minutes to complete.

If you are interested in participating, click on the survey link below. Proceeding forward to the survey implies that you consent to participate in this research study.

If you have any questions about this study, you can reply to this email or contact a member of the study team at [sri*********@duke.edu](mailto:sri*********@duke.edu) (or) 678-***-****.

Thank you for your time and consideration.

Sincerely,

Sriram Venkatesan, MS4

Study Team

Division of Emergency Medicine

Duke University School of Medicine

This study has been approved by the Duke Health Institutional Review Board, Protocol Number: Pro00106210.

**DSM-5 Definition of a Trauma**

Exposure to actual or threatened a) death, b) serious injury, c) sexual violation, in one or more of the following ways:

1. Directly experiencing the traumatic event(s)
Examples of directly experienced traumatic events: combat, life threatening accident (e.g., plane crash, motor vehicle accident), violent physical/sexual assault (in childhood or adulthood), torture, incarceration as a prisoner of war or in a concentration camp, natural or man-made disaster (e.g., earthquake, fire, hurricane, flood), robbery, stabbing/shooting, being diagnosed with a life-threatening illness.

2. Witnessing, in person, the traumatic event(s) as they occurred to others

Examples of witnessed traumatic events: observing death or injury of another person due to assault, war, or disaster, unexpectedly seeing a dead body or body parts.

3. Learning that the traumatic event(s) occurred to a close family member or close friend; cases of actual or threatened death must have been violent or accidental
Examples of traumatic events confronted with or learned about: learning of a family member’s (or friend’s) sudden, unexpected death, or learning that one’s child has a life-threatening disease.

4. Experiencing repeated or extreme exposure to aversive details of the traumatic event(s). This does not apply to exposure through electronic media, television, movies, or pictures, unless this exposure is work related

Examples of repeated or extreme exposure to aversive details of traumatic events: first responders collecting human remains; police officers repeatedly exposed to details of child abuse

1. In this study, we are specifically focusing on incidents such as those mentioned above that you faced in the emergency department since the start of the COVID-19 pandemic in the US. Based on that characterization, would you consider yourself a victim to your trauma?

1. Yes
2. No

   2. Have you ever been previously diagnosed/treated with PTSD?
3. Yes
4. No

3. Have you ever been previously diagnosed/treated for depression or any other mental health disease?

1. Yes
2. No

4. Are you currently undergoing any mental health services, including counseling, support groups or medications?

1. Yes
2. No

5. What resources are available at your institution for mental health?

1. On-site Private Counsellor
2. Confidential Counselling hotline
3. (Free Text-Type-in)

The Coronavirus (COVID-19) pandemic has been present in the United States since February, 2020. During this time period:

Select ONE response for each of the following questions:

| **S. No** | **Question** | **0**  **Not at all** | **1**  **A little (once a week or less)** | **2**  **Somewhat (2-3 times a week)** | **3**  **Very much (4-5 times a week)** | **4**  **Severe (>6 times/week)** |
| --- | --- | --- | --- | --- | --- | --- |
| 1 | Have you had **unwanted distressing memories** about the trauma? |  |  |  |  |  |
| 2 | Have you been having **bad dreams or nightmares** related to the trauma? |  |  |  |  |  |
| 3 | Have you had the experience of feeling as if the trauma were **actually happening again**? |  |  |  |  |  |
| 4 | Have you been very **EMOTIONALLY upset** when reminded of the trauma? |  |  |  |  |  |
| 5 | Have you had **PHYSICAL reactions** when reminded of the trauma (e.g., sweating, heart racing)? |  |  |  |  |  |
| 6 | Have you been making efforts to **avoid thoughts or feelings related to the trauma**? |  |  |  |  |  |
| 7 | Have you been making efforts to **avoid activities, situations, or places that remind you of the trauma** or that feel more dangerous since the trauma? |  |  |  |  |  |
| 8 | Are there any important **parts of the trauma that you cannot remember**? |  |  |  |  |  |
| 9 | Have you been viewing yourself, others, or the **world in a more negative way** (e.g., “I can’t trust people,” “I’m a weak person”)? |  |  |  |  |  |
| 10 | Have you **blamed yourself for the trauma** or for what happened afterwards? Have you blamed others that did not directly cause the event for the trauma or what happened afterwards? |  |  |  |  |  |
| 11 | Have you had **intense negative feelings** such as fear, horror, anger, guilt or shame? |  |  |  |  |  |
| 12 | Have you **lost interest** in activities you used to do? |  |  |  |  |  |
| 13 | Have you **felt detached** or cut off from others? |  |  |  |  |  |
| 14 | Have you had **difficulty experiencing positive feelings**? |  |  |  |  |  |
| 15 | Have you been **acting more irritable or aggressive**? |  |  |  |  |  |
| 16 | Have you been **taking more risks** or doing things that might cause you or others harm (e.g., driving recklessly, taking drugs, having unprotected sex)? |  |  |  |  |  |
| 17 | Have you been **overly alert or on-guard** (e.g., checking to see who is around you, etc.)? |  |  |  |  |  |
| 18 | Have you been **jumpier or more easily startled**? |  |  |  |  |  |
| 19 | Have you had **difficulty concentrating**? |  |  |  |  |  |
| 20 | Have you had **difficulty falling or staying asleep**? |  |  |  |  |  |

21. In your view, what are the triggers of PTSD at your workplace:

1. Shift acuity
2. Fear of getting sick
3. Fear of family/friends getting sick
4. Lack of PPE
5. Lack of standard for treating COVID-19
6. Overcrowding
7. Free Text (Type-in)

**Demographic Information:**

1. **What position do you currently hold?**

    a) Emergency Medicine Resident
    b) Emergency Medicine Board-Certified Physician
    c) Emergency Medicine Board-Eligible Physician

d) Non-EM board certified Physician working in an EM setting

1. **What is your age?**

a) 20-35
 b) 35-50
 c) 50-65
 d) >65

1. **What is your gender?**

    a) Male
    b) Female
    c) Transgender
    d) Non-Binary

e) Prefer not to answer

4. **What is your ethnicity?** a) Caucasian/White
 b) African American/Black
 c) Hispanic/Latino
 d) Asian
 e) Pacific Islander

f) Prefer Not to Answer

5. Which state do you currently practice in?

[Dropdown of all US States and Territories]

6. **Describe the population served by your practice?**

 a) Urban
 b) Suburban
 c) Small City
 d) Rural
 e) Geographically isolated/Remote

7. **What is your practice setting?**

 a) Level 1 Trauma Center
 b) Level 2 Trauma Center
 c) Level 3/4 Trauma Center

d) Urgent Care

e) Other (Free Text)

8. **Have you ever been a member of the US Armed Forces/Military?**

 a) Yes
 b) No

9. **If yes for Question #7, did you serve abroad?**

1. Yes
2. No

The following questions ask for your views about your health. Answer every question, by selecting the answer most appropriate. If you are unsure about how to answer a question, please give the best answer you can.

1. Overall, how would you rate your health during the **past 4 weeks**?
2. Excellent
3. Very Good
4. Good
5. Fair
6. Poor
7. Very Poor
8. During the **past 4 weeks**, how much did physical health problems limit your physical activities (such as walking or climbing stairs)?
9. Not at all
10. Very little
11. Somewhat
12. Quite a lot
13. Could not do physical activities
14. During the **past 4 weeks**, how much difficulty did you have doing your daily work, both at home and away from home, because of your physical health?
15. Not at all
16. Very little
17. Somewhat
18. Quite a lot
19. Could not do physical activities
20. How much bodily pain have you had during the **past 4 weeks**?
21. None
22. Very mild
23. Mild
24. Moderate
25. Severe
26. Very severe
27. During the **past 4 weeks**, how much energy did you have?
28. Very much
29. Quite a lot
30. Some
31. A little
32. None
33. During the **past 4 weeks**, how much did your physical health or emotional problems limit your usual social activities with family or friends?
34. Not at all
35. Very little
36. Somewhat
37. Quite a lot
38. Could not do social activities
39. During the **past 4 weeks**, how much have you been bothered by **emotional problems** (such as feeling anxious, depressed or irritable)?
40. Not at all
41. Slightly
42. Moderately
43. Quite a lot
44. Extremely
45. During the **past 4 weeks**, how much did personal or emotional problems keep you from doing your usual work, school or other daily activities?
46. Not at all
47. Very little
48. Somewhat
49. Quite a little
50. Could not do daily activities

Thank you for completing this survey!
